# Supplementary material for: Endothelial deletion of SHP2 suppresses tumor angiogenesis and promotes vascular normalization
Source: Nat Commun. 2021 Nov 2;12:6310. doi: 10.1038/s41467-021-26697-8 (PMC8564544; doi:10.1038/s41467-021-26697-8)
Supplement: Supplementary file 1 — Supplementary Information [file 41467_2021_26697_MOESM1_ESM.pdf]

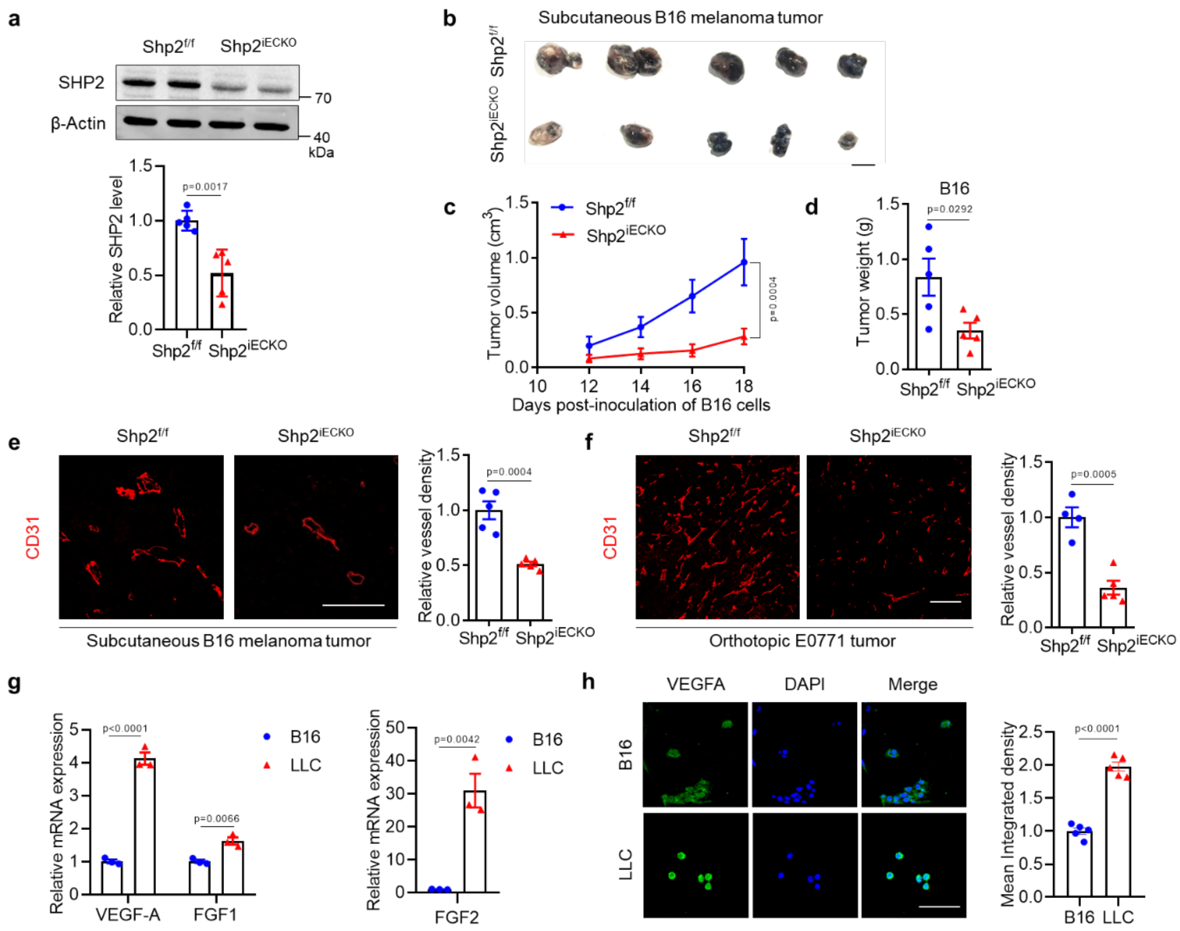

Supplementary Fig. 1. Shp2 deletion in endothelial cells reduces tumor angiogenesis. **a** Western blot for SHP2 in lung endothelial cells from Shp2$^{f/f}$ and Shp2^{iECKO} mice. Quantitative data were shown as mean ± SEM for five independent experiments. p-value was shown and generated by using the two-tailed Student's *t*-test. **b** Images for subcutaneous B16 melanoma tumors in Shp2$^{f/f}$ (n = 5) and Shp2^{iECKO} (n = 5) mice. Scale bar: 10 mm. **c, d** The volumes (c) and weights (d) for B16 tumors in Shp2$^{f/f}$ (n = 5) and Shp2^{iECKO} (n = 5) mice. Tumor weights were measured 18 days after cancer cell injection. Quantitative data were shown as mean ± SEM. p-values were shown and generated by two-way ANOVA with Tukey's post hoc test or by using the two-tailed Student's *t*-test. **e, f** Immunofluorescence staining for CD31 in subcutaneous B16 melanoma tumors (n=5 for each group) and orthotopic E0771 tumors (n=4 for Shp2$^{f/f}$ group and n=5 for Shp2^{iECKO} group). Quantitative data were measured by using the Image J software and shown as mean ± SEM. p-values were shown and generated by using the two-tailed Student's *t*-test. Scale

bar: 100 μm. **g, h** qPCR for VEGFA, FGF1 and FGF2 and immunofluorescence staining for VEGFA (h) in B16 and LLC cancer cells by Z-staking. Quantitative data were shown as mean ± SEM for five independent experiments. p-values were shown and generated by using the two-tailed Student's *t*-test. Scale bar: 20 μm. Source data are provided as a Source Data file.

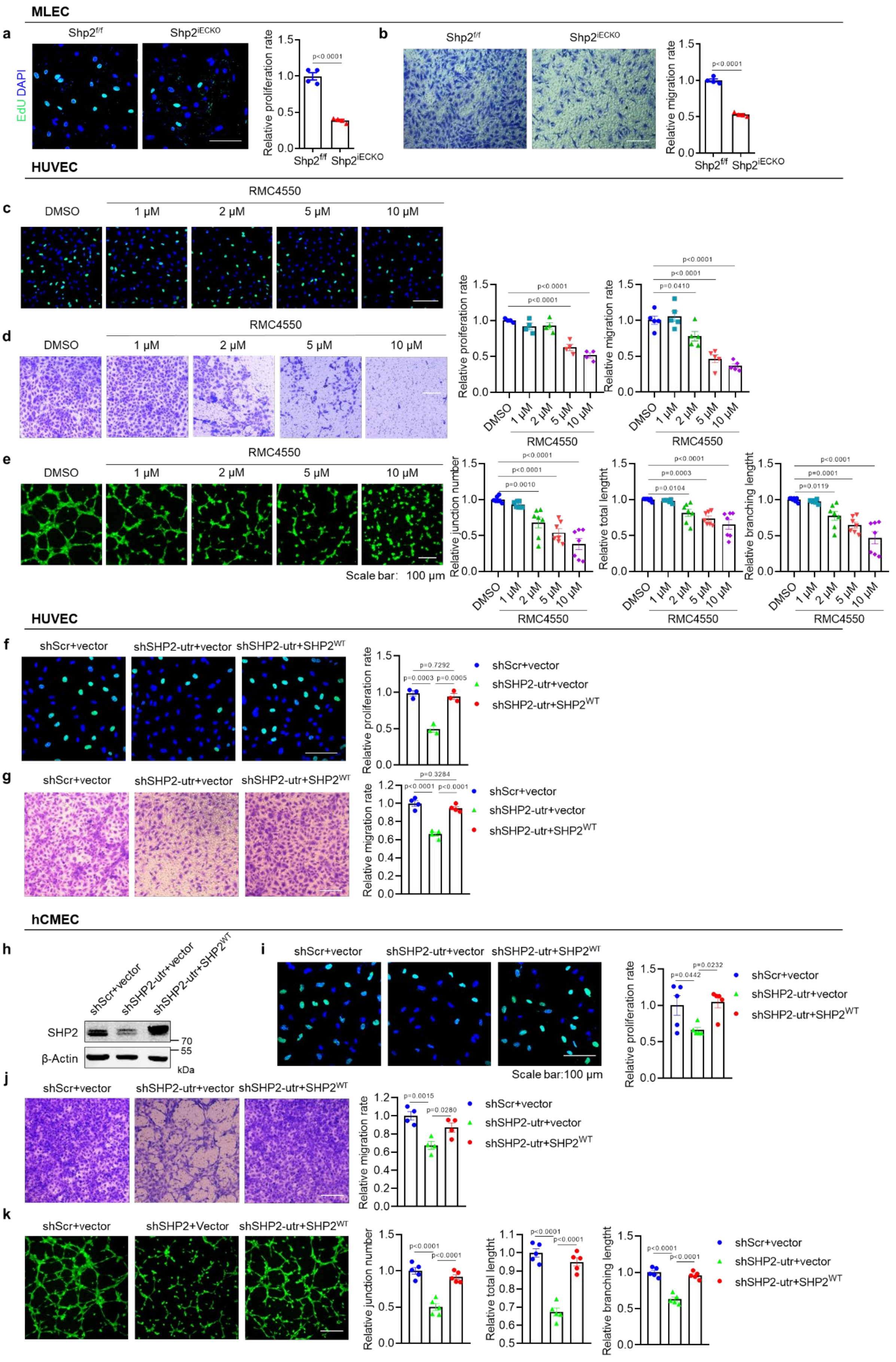
MLEC
a
Shp2f/f
Shp2iECKO
EdU DAPI
Relative proliferation rate
p<0.0001
b
Relative migration rate
HUVEC
c
RMC4550
DMSO
1 μM
2 μM
5 μM
10 μM
d
e
Scale bar: 100 μm
Relative junction number
Relative total lenght
Relative branching lenght
p=0.0410
p=0.0010
p=0.0104
p=0.0003
p=0.0119
f
shScr+vector
shSHP2-utr+vector
shSHP2-utr+SHP2WT
p=0.7292
p=0.0003
p=0.0005
g
p=0.3284
hCMEC
h
SHP2
β-Actin
70
55
kDa
i
p=0.0442
p=0.0232
Scale bar:100 μm
j
p=0.0015
p=0.0280
k
shSHP2+Vector

Supplementary Fig. 2. Inhibition or knockdown of SHP2 impairs endothelial function in vitro.

a, **b** EdU incorporation assay (a) and transwell migration assay (b) for mouse lung endothelial cells (MLECs) isolated from $Shp2^{f/f}$ (n = 3) and $Shp2^{iECKO}$ (n = 3) mice. Images were analyzed by the Image J software and quantitative data were shown as mean ± SEM. p-values were shown and generated by using the two-tailed Student's t-test. Scale bar: 100 μm. **c-e** Cell proliferation measured by EdU incorporation assay (c, n=4), cell migration measured by transwell migration assay (d, n=5) and in vitro tube formation (e, n=7) of HUVECs treated with SHP2 inhibitor (RMC4550, 5 μM, 24 h). Junction numbers, tube lengths, and branching lengths were measured by using the Image J software and shown as mean ± SEM. p-values were shown and generated by using one-way ANOVA with multi-comparisons. Scale bar: 100 μm. **f, g** Cell proliferation measured by EdU incorporation assay (f, n=3) and cell migration measured by transwell migration assay (g, n=4) in SHP2-knockdown HUVECs with SHP2 re-expression. Images were analyzed by the Image J software and quantitative data were shown as mean ± SEM. p-values were shown and generated by using one-way ANOVA with multi-comparisons. Scale bar: 100 μm. **h** Western blot for SHP2 expression in SHP2-knockdown hCMECs with SHP2 re-expression. β-Actin was used as a loading control. Results were repeated for three independent experiments. **i-k** Cell proliferation measured by EdU incorporation assay (i, n=5), cell migration measured by transwell migration assay (j, n=4) and tube formation in vitro (k, n=5) in SHP2-knockdown hCMEC with SHP2 re-expression. Junction numbers, tube lengths, and branching lengths were measured by using the Image J software and shown as mean ± SEM. p-values were shown and generated by using one-way ANOVA with multi-comparisons. Scale bar: 100 μm. Source data are provided as a Source Data file.

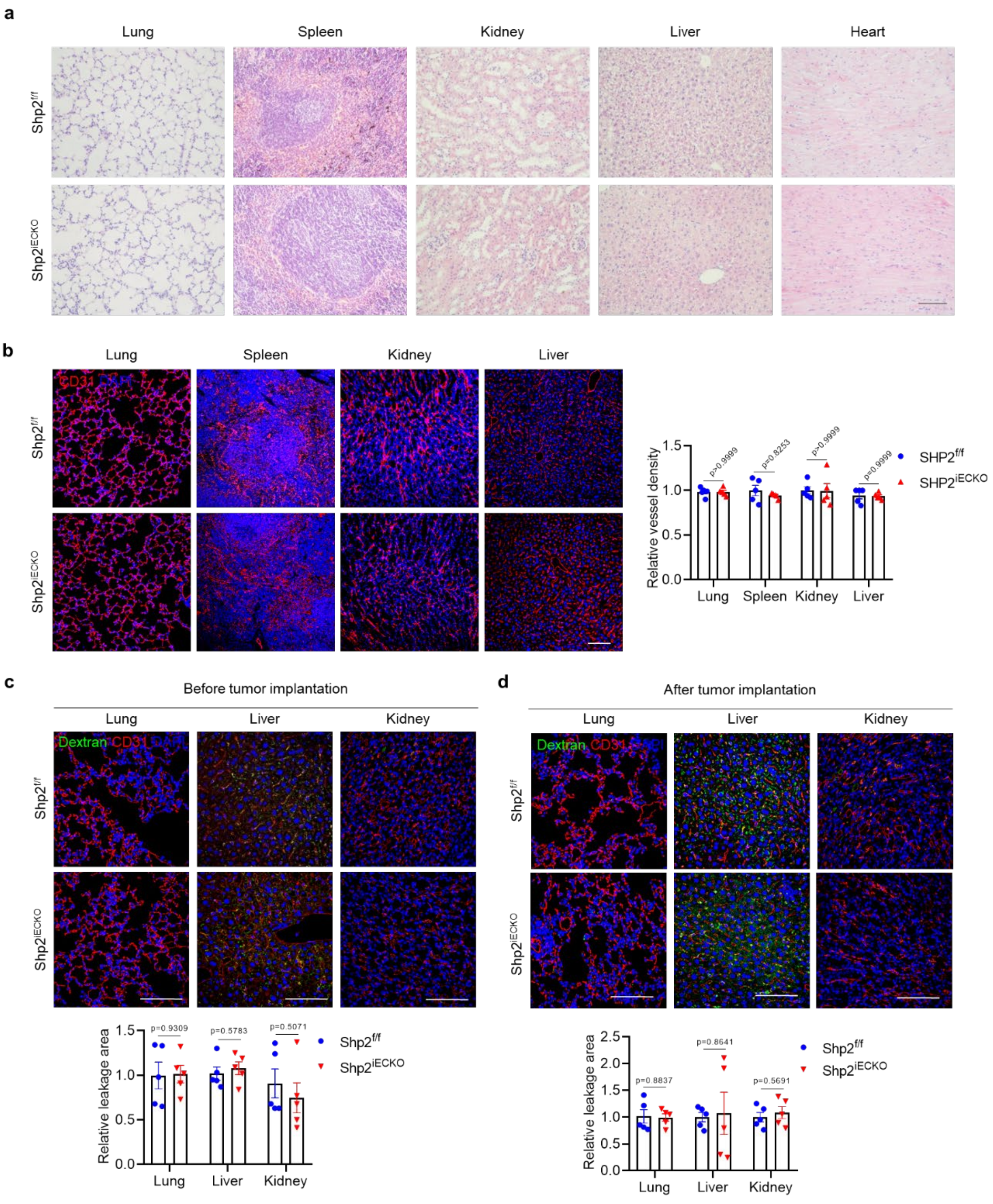

Supplementary Fig. 3. Shp2 deletion in adult mouse endothelia does not affect microvasculature in various organs. **a** H&E staining images showing histological morphology of lungs, spleens, kidneys, livers and hearts of $Shp2^{f/f}$ (n = 5) and $Shp2^{iECKO}$ (n = 5) mice. Scale bar: 100 μm. **b** Immunofluorescence images of $CD31^{+}$ vessels in lungs, spleens, kidneys and livers in $Shp2^{f/f}$ (n = 5) and $Shp2^{iECKO}$ (n = 5) mice. Quantitative

data were shown as mean ± SEM. p-values were shown and generated by using the two-tailed Student's *t*-test. Scale bar: 100 µm. **c, d** Immunofluorescent images of FITC-dextran in lungs, livers and kidneys in $Shp2^{f/f}$ (n = 5) and $Shp2^{iECKO}$ (n = 5) mice before (c) or after (d) tumor implantation. Quantitative data were shown as mean ± SEM. p-values were shown and generated by using the two-tailed Student's *t*-test. Scale bar: 100 µm. Source data are provided as a Source Data file.

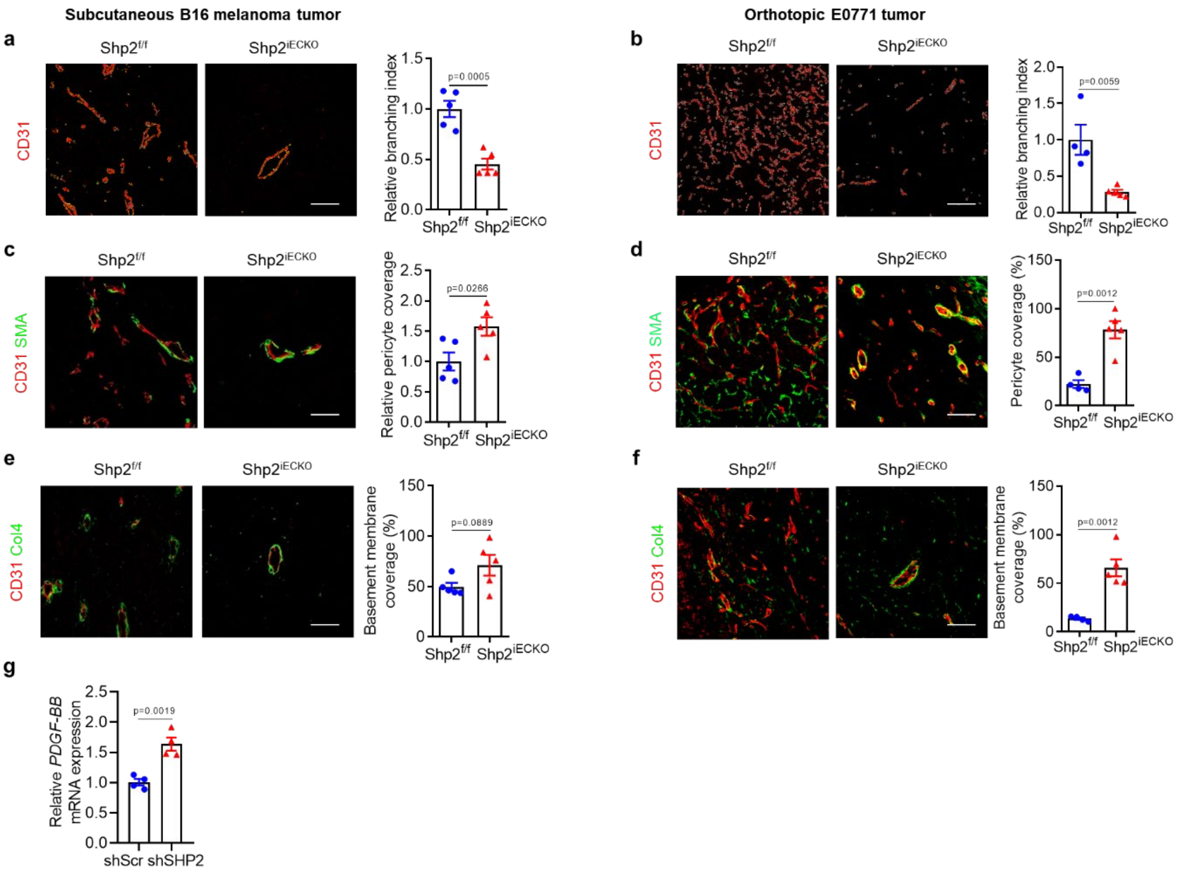

Supplementary Fig. 4. Shp2 deletion in endothelial cells promotes tumor vessel normalization. a, b Immunofluorescence staining for CD31 in subcutaneous B16 melanoma tumor and orthotopic E0771 tumors in Shp2$^{f/f}$ (n = 5 for B16 tumors, n = 4 for E0771 tumors) and Shp2^{iECKO} (n = 5 for B16 tumors, n = 5 for E0771 tumors) mice. Vessel branching index were measured by using Image J software and quantitative data were shown as mean ± SEM. p-values were shown and generated by using the two-tailed Student's *t*-test. Scale bar: 100 μm. **c, d** Immunofluorescence staining for αSMA in subcutaneous B16 melanoma tumors and orthotopic E0771 tumors from Shp2$^{f/f}$ (n = 5 for B16 tumors, n = 4 for E0771 tumors) and Shp2^{iECKO} (n = 5 for B16 tumors, n = 5 for E0771 tumors) mice. Pericyte coverage were measured by using the Image J software and quantitative data were shown as mean ± SEM. p-values were shown and generated by using the two-tailed Student's *t*-test. Scale bar: 100 μm. **e, f** Immunofluorescence staining for Collagen IV(Col4) in subcutaneous B16 melanoma tumors and orthotopic E0771 tumors from Shp2$^{f/f}$ (n = 5 for B16 tumors, n = 4 for E0771 tumors) and Shp2^{iECKO} (n = 5 for B16 tumors, n = 5 for E0771 tumors) mice. Basement membrane coverage were measured by

using the Image J software and quantitative data were shown as mean ± SEM. p-values were shown and generated by using the two-tailed Student's *t*-test. Scale bar: 100 μm. **g** qPCR for *PDGF-BB* in SHP2-knockdown endothelial cells. Quantitative data were shown as mean ± SEM for four independent experiments. p-value was shown and generated by using the two-tailed Student's t-test. Source data are provided as a Source Data file.

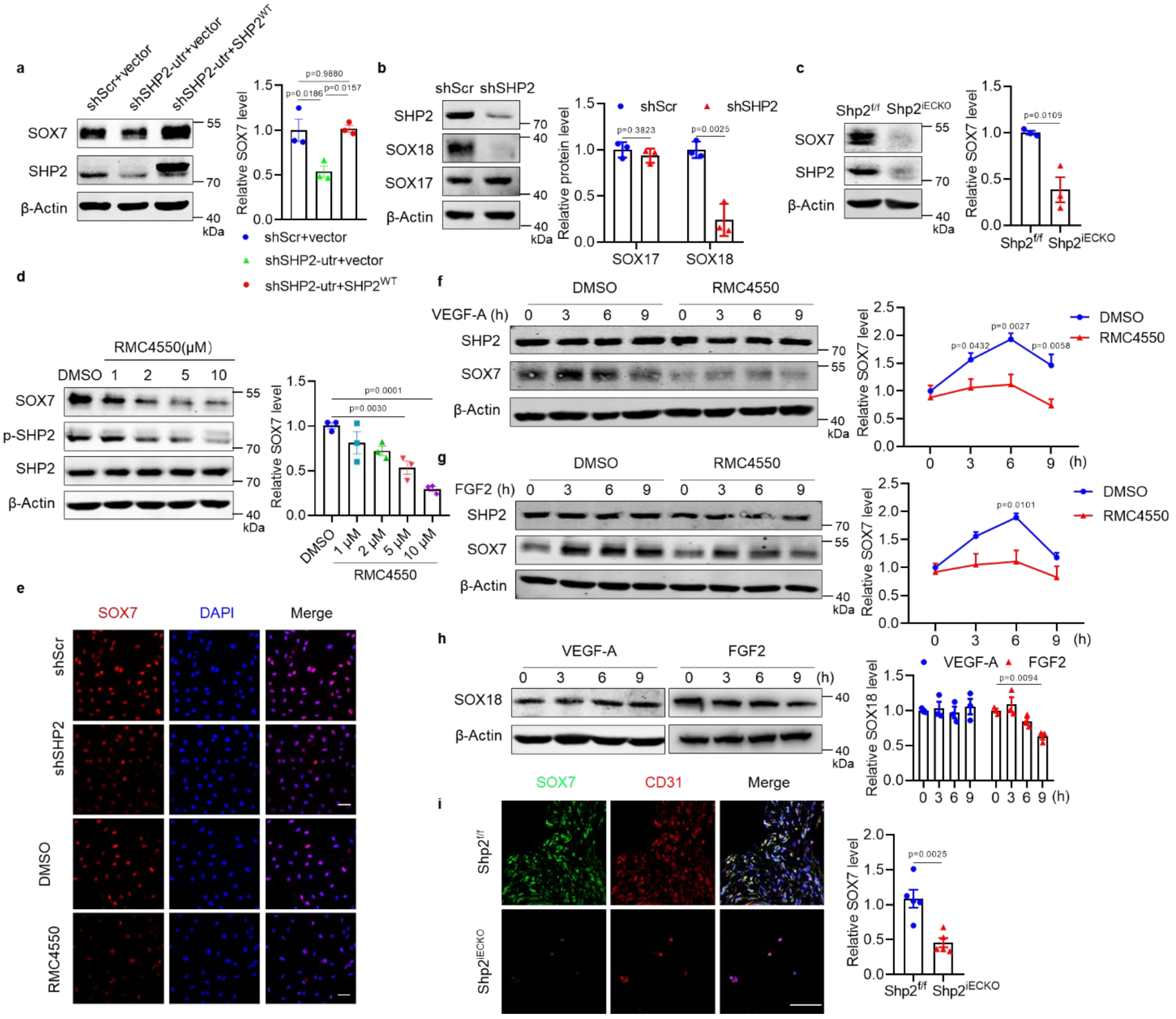

Supplementary Fig. 5. SHP2 regulates c-Jun signaling and SOX7 expression.

a Western blot for SOX7 in SHP2-knockdown hCMECs with SHP2 re-expression. β-Actin was used as a loading control. Quantitative data were shown as mean ± SEM for three independent experiments. p-values were shown and generated by one-way ANOVA with multi-comparisons. **b-d** Western blot for SOX7, SOX17, and SOX18 in SHP2-knockdown HUVECs (b, n=3), or SOX7 in lung endothelial cells isolated from $Shp2^{f/f}$ and $Shp2^{iECKO}$ mice (c, n=3), or HUVECs treated with various concentrations of SHP2 inhibitor RMC4550 for 24 h (d, n=3). β-Actin was used as a loading control. Quantitative data were shown as mean ± SEM. p-values were shown and generated by one-way ANOVA with multi-comparisons or using the two-tailed Student's *t*-test. **e** Immunofluorescence staining

for SOX7 in SHP2-knockdown HUVECs. DAPI labeled nuclei. Scale bar: 50 μm. Results were repeated for three independent experiments. **f, g** Western blot for SOX7 and SHP2 for HUVECs treated with RMC4550 (5 μM, 24 h) and VEGF (f, 10 ng/ml, n=3) or FGF2 (g, 10 ng/ml , n=3). β-Actin was used as a loading control. Quantitative data were shown as mean ± SEM. p-values were shown and generated by two-way ANOVA with Bonferroni's multiple comparisons test. **h** Western blot for SOX18 in VEGF or FGF2 treated HUVECs. β-Actin was used as a loading Quantitative data were shown as mean ± SEM for three independent experiments. p-values were shown and generated by one-way ANOVA with multi comparisons. **i** Immunofluorescence staining for SOX7 in the plugs in $Shp2^{f/f}$ (n = 5) and $Shp2^{iECKO}$ (n = 5) mice. CD31 labeled endothelial cells. Quantitative data were measured by using the ImageJ software and shown as mean ± SEM. p-value was shown and generated by using the two-tailed Student's t-test. Scale bar: 100 μm . Source data are provided as a Source Data file.

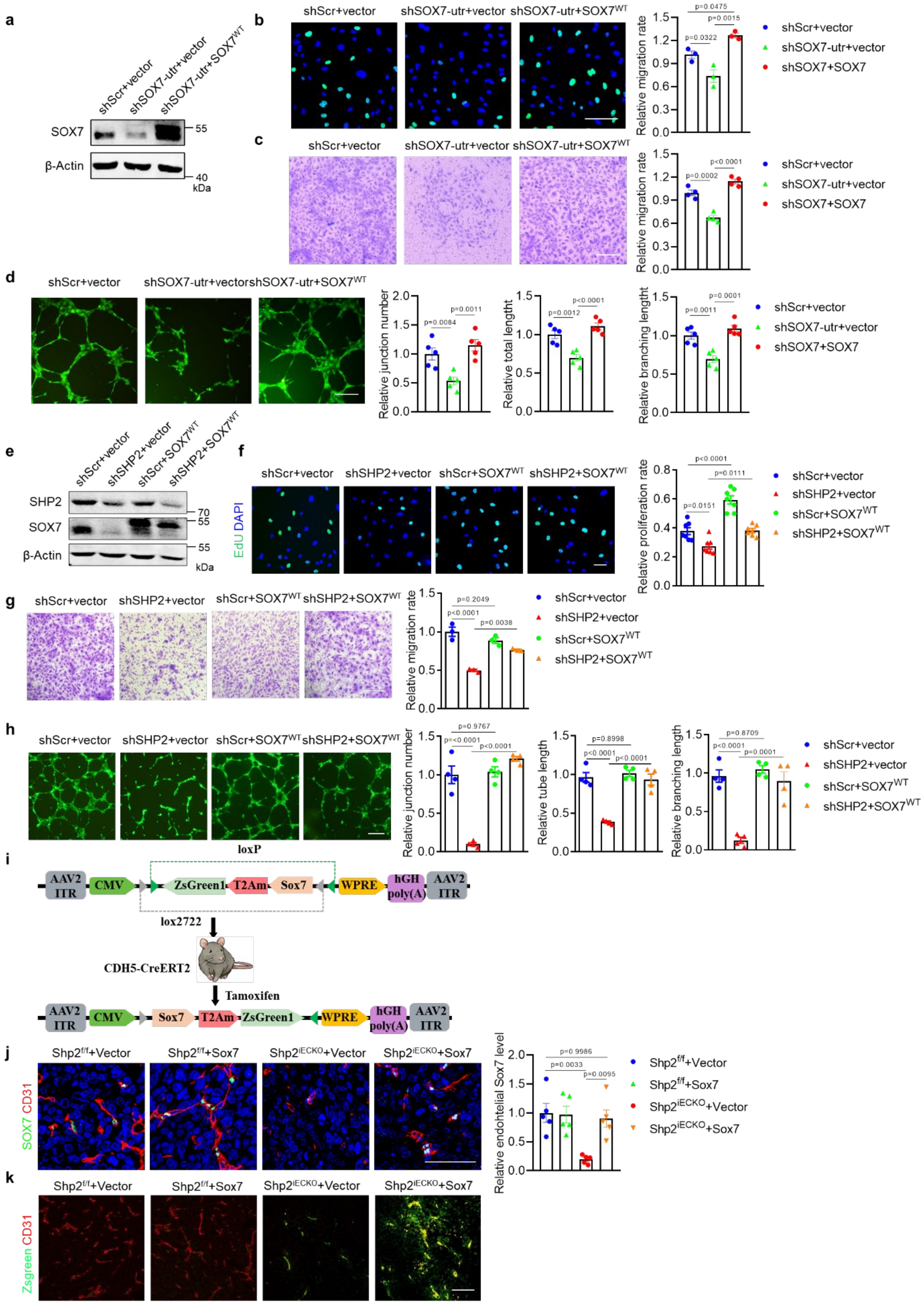
a
shScr+vector
shSOX7-utr+vector
shSOX7-utr+SOX7WT
SOX7
β-Actin
55
40
kDa
b
shScr+vector
shSOX7-utr+vector
shSOX7-utr+SOX7WT
Relative migration rate
p=0.0475
p=0.0015
p=0.0322
shScr+vector
shSOX7-utr+vector
shSOX7+SOX7
c
p<0.0001
p=0.0002
d
Relative junction number
Relative total lenght
Relative branching lenght
p=0.0084
p=0.0011
p=0.0012
p<0.0001
p=0.0011
p=0.0001
e
shSHP2+vector
shScr+SOX7WT
shSHP2+SOX7WT
SHP2
SOX7
70
f
EdU DAPI
Relative proliferation rate
p<0.0001
p=0.0111
p=0.0151
g
p=0.2049
p<0.0001
p=0.0038
h
Relative tube length
p=0.9767
p=0.8998
p=0.8709
i
loxP
lox2722
AAV2 ITR
CMV
ZsGreen1
T2Am
Sox7
WPRE
hGH poly(A)
CDH5-CreERT2
Tamoxifen
j
Shp2f/f+Vector
Shp2f/f+Sox7
Shp2iECKO+Vector
Shp2iECKO+Sox7
SOX7 CD31
Relative endohtelial Sox7 level
p=0.9986
p=0.0033
p=0.0095
k
Zsgreen CD31

Supplementary Fig. 6. SOX7 functions as a key downstream effector for SHP2 in regulating angiogenesis. a Western blot for SOX7 in SOX7-knockdown HUVECs with SOX7 re-expression. Results were repeated for three independent experiments. **b-d** Cell proliferation measured by EdU incorporation assay (b, n=3), cell migration measured by transwell migration assay (c, n=4) and tube formation in vitro (d, n=5) of HUVECs. Junction numbers, tube lengths, and branching lengths were measured by using the Image J software and shown as mean ± SEM. p-values were shown and generated by using one-way ANOVA with multi-comparisons. Scale bar: 100 μm. **e** Western blot for SOX7 in SHP2-knockdown HUVECs with SOX7 re-expression. Results were repeated for three independent experiments. **f-h** Cell proliferation measured by EdU incorporation assay (f, n=7), cell migration measured by transwell migration assay (g, n=3) and tube formation in vitro (h, n=4) of HUVECs. Junction numbers, tube lengths, and branching lengths were measured by using the Image J software and shown as mean ± SEM. p-values were shown and generated by using one-way ANOVA with multi-comparisons. Scale bar: 100 μm. **i** Strategy to specifically express SOX7 in tumor endothelial cells. A cassette to express SOX7 and ZsGreen was inserted between the two pairs of loxp sequences. Due to the opponent direction of Sox7 cDNA and CMV promotor, no Sox7 was transcripted without any intervention. Tumors was infected with AAV by intratumoral multi-point injection. Tamoxifen was used to induce Cre expression in endothelial cells in $Shp2^{iECKO}$ mice. Cre recombinase functioned to flip the Sox7 cassette, and thereafter SOX7 was shown under CMV promotor in tumor endothelial cells in $Shp2^{iECKO}$ mice. **j** Representative images showing SOX7 expression (green) in CD31-positive vessels (red) in LLC tumors (n=5 for each group). Endothelial SOX7 were analyzed by the Image J software and Quantitative data were shown as mean ± SEM. p-values were shown and generated by one-way ANOVA with Tukey's post hoc test. Scale bar: 50 μm. **k** Representative images for GFP in LLC tumors in $Shp2^{iECKO}$ mice**.** GFP was not shown in $Shp2^{f/f}$ mice without Cre recombinase (n=5 for each group). Scale bar: 100 μm. Source data are provided as a Source Data file.

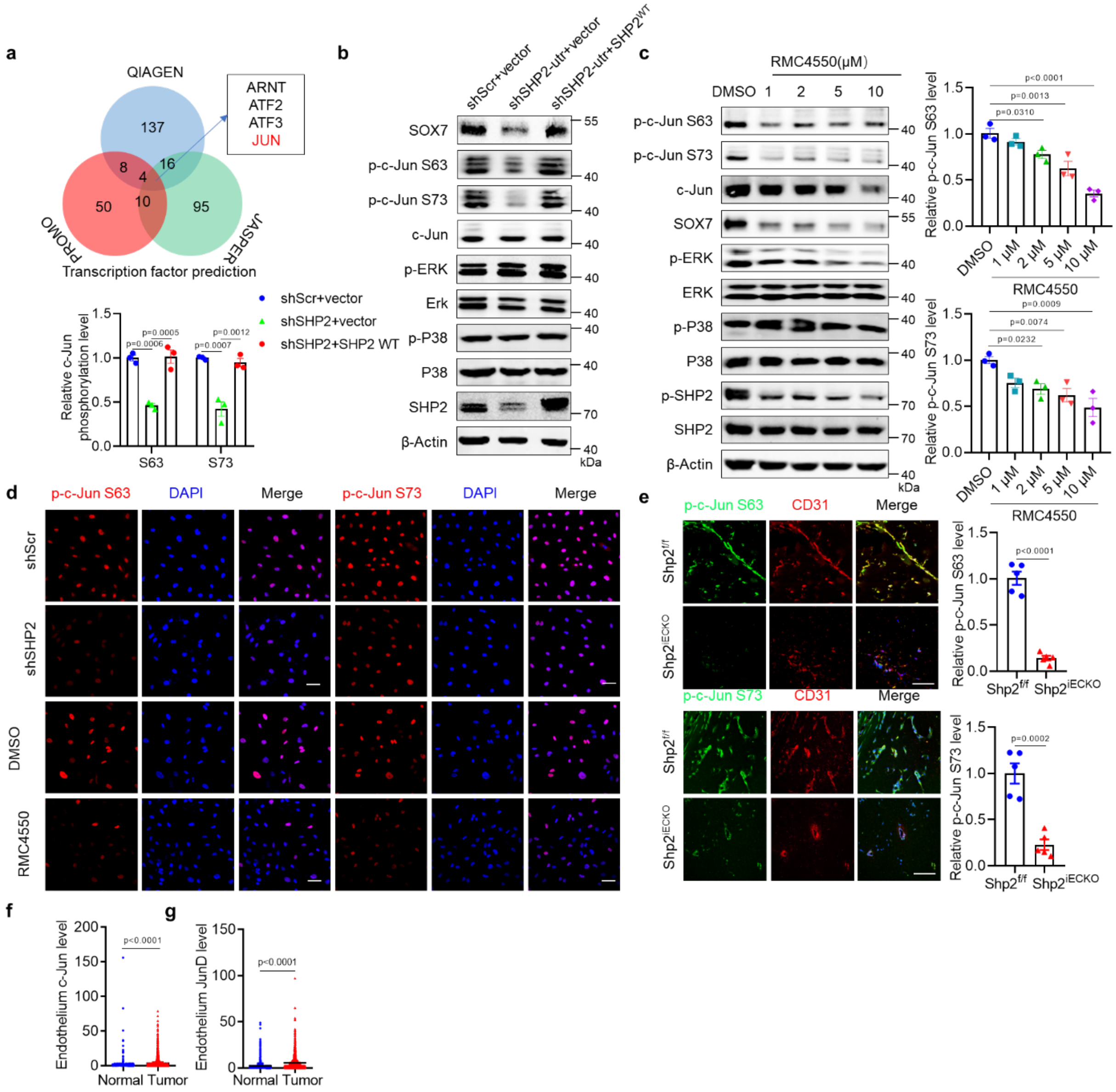

Supplementary Fig. 7. c-Jun is critical for SHP2 in controlling SOX7 expression. a Four transcriptional factors including c-Jun were predicted for SOX7 in all three databases. **b, c** Western blot for c-Jun, ERK, and p-38 and associated phosphorylated forms in SHP2-knockdown hCMECs or HUVECs treated with SHP2 inhibitor (RMC4550) for 24 h. β-Actin was used as a loading control. Quantitative data were shown as mean ± SEM for three independent experiments. p-values were shown and generated by one-way ANOVA with multi-comparisons or the two-tailed Student's *t*-test. **d** Immunofluorescence staining for phosphorylated c-Jun (Ser 63 and Ser 73) in SHP2-knockdown HUVECs or HUVECs treated with SHP2 inhibitor (RMC4550, 24h). DAPI was used to label nuclei. Scale bar:

50 μm. Results were repeated for three independent experiments. **e** Immunofluorescence staining for phosphorylated c-Jun (Ser 63 and Ser 73) in the plugs in $Shp2^{f/f}$ (n = 5) and $Shp2^{iECKO}$ (n = 5) mice. CD31 labeled endothelial cells. Quantitative data were measured by using the ImageJ software and shown as mean ± SEM. p-value was shown and generated by using the two-tailed Student's *t*-test. Scale bar: 100 μm. **f, g** mRNA for c-Jun (f) and JunD (g) were extracted from GSE118904 database to show increased expression in tumor endothelial cells compared with that in normal endothelial cells. n = 1000 cells, p-values were shown and generated by using the two-tailed Student's *t*-test. Source data are provided as a Source Data file.

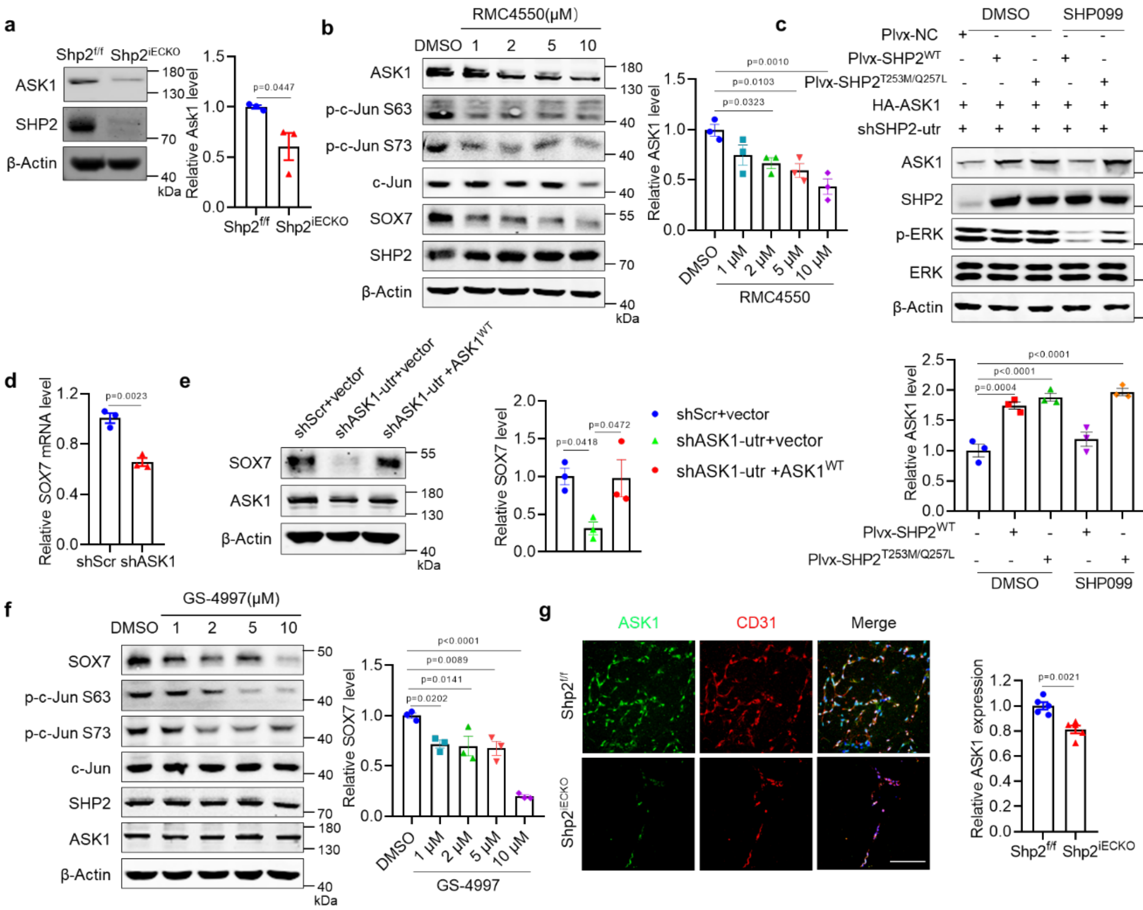

Supplementary Fig. 8. SHP2 positively regulates c-Jun/SOX7 signaling axis by stabilizing ASK1. a Western blot for ASK1 in MLECs isolated from Shp2$^{f/f}$ and Shp2^{iECKO} mice (n = 3). β-Actin was used as a loading control. Quantitative data were shown as mean ± SEM. p-value was shown and generated by using the two-tailed Student's *t*-test. **b** Western blot for ASK1, c-Jun, phosphor-c-Jun, SOX7, and SHP2 in HUVECs treated with SHP2 inhibitor (RMC4550, 5 μM) for 24 h. β-Actin was used as a loading control. Quantitative data were shown as mean ± SEM for three independent experiments. p-values were shown and generated by one-way ANOVA with multi-comparisons. **c** Western blot for ASK1, ERK, p-ERK, and SHP2 in HUVECs. SHP2^{WT} or SHP2$^{T253M/Q257L}$ was shown in SHP2-knockdown HUVECs and cells were treated with SHP2 inhibitor (SHP099, 5 μM) for 24 h. β-Actin was used as a loading control. **d-f** qPCR (d) and Western blot (e, f) for SOX7 in ASK1-knockdown or ASK1 inhibitor GS-4997 (24 h) treated HUVECs. β-Actin was used as a loading control. Quantitative data were shown as mean ± SEM for three independent experiments. p-values were shown and generated by using the two-tailed Student's t-test or one-way ANOVA with multi-comparisons. **g** Immunofluorescence

staining for ASK1 in the plugs in $Shp2^{f/f}$ (n = 5) and $Shp2^{iECKO}$ (n = 5) mice. CD31 labeled endothelial cells. Quantitative data were measured by using the Image J software and shown as mean ± SEM. p-value was shown and generated by using the two-tailed Student's *t*-test. Scale bar: 100 μm. Source data are provided as a Source Data file.

Supplementary Table 1: Antibodies

Antibody	Cat number	Dilution	Source	Company
Anti-ASK1	NB100-81788	WB: 1:1000<br>IF: 1:100	Rabbit	Novus Biologicals
Anti-SHP2	#3397	WB: 1:1000	Rabbit	Cell Signaling Technology
Anti-SHP2	sc-7384	IF: 1:50	Mouse	Santa Cruz
Anti-p-SHP2	ab62322	WB: 1:1000<br>IF: 1:100	Rabbit	Abcam
Anti-SOX7	23925-1-AP	WB: 1:1000<br>IF: 1:100	Rabbit	Proteintech
Anti-SOX17	24903-1-AP	WB: 1:1000	Rabbit	Proteintech
Anti-p-c-Jun S63	#91952	WB: 1:1000<br>IF: 1:100	Rabbit	Cell Signaling Technology
Anti-p-c-Jun S73	#3270	WB: 1:1000<br>IF: 1:100	Rabbit	Cell Signaling Technology
Anti-c-Jun	db2622	WB: 1:1000	Rabbit	Diagbio
Anti-p-ERK	#9101	WB: 1:1000	Rabbit	Cell Signaling Technology
Anti-ERK	#4695	WB: 1:1000	Rabbit	Cell Signaling Technology
Anti-p-p38	#4511	WB: 1:1000	Rabbit	Cell Signaling Technology
Anti-p38	#8690	WB: 1:1000	Rabbit	Cell Signaling Technology
Anti-β-Actin	M1210-2	WB: 1:1000	Mouse	Huabio
Anti-Vegfa	19003-1-AP	WB: 1:1000	Rabbit	Proteintech
Anti-HA	AF5057	WB: 1:1000	Mouse	Beyotime
Anti-MYC	AF5054	WB: 1:1000	Mouse	Beyotime
Anti-CD31	AF3628	IF: 1:200	Goat	R&D system
Anti-CD31	ab28364	IF: 1:200	Rabbit	Abcam
Anti-collagen IV	ab236640	IF: 1:200	Rabbit	Abcam

Anti-α-SMA	#19245	IF: 1:200	Rabbit	Cell Signaling Technology
Anti-CD11b	101213	IF: 1:200	Rat	Biolegend
800CW Goat anti-Rabbit(WB)	925-32211	1:5000	Goat	LI-COR
680RD Goat anti-Mouse(WB)	925-68070	1:5000	Goat	LI-COR
Donkey anti-Mouse, Alexa Fluor 488	A-21202	IF: 1:200	Donkey	ThermoFisher
Donkey anti-Rabbit, Alexa Fluor 488	A-21206	IF: 1:200	Donkey	ThermoFisher
Donkey anti-Rabbit, Alexa Fluor 555	A-31572	IF: 1:200	Donkey	ThermoFisher
Donkey anti-Goat, Alexa Fluor 647	A-21447	IF: 1:200	Donkey	ThermoFisher
AffiniPure Donkey Anti-Goat, Alexa Fluor 488	705-545-103	IF: 1:200	Donkey	Jackson Immuno Research
AffiniPure Donkey Anti-Goat, Cyanine Cy3	705-165-103	IF: 1:200	Donkey	Jackson Immuno Research

Supplementary Table 2 qPCR primers for human

**Primer Name**	**Primer sequence 5'-3'**
VEGFA F	GATGAGCTTCCTACAGCACAACAA
VEGFA R	TTTCGTTTTTGCCCCTTTCC
VEGFR1 F	GGGACAGTAGAAAGGGCTTCATC
VEGFR1 R	TGGGCGTGGTGTGCTTATTT
VEGFR2 F	CGCAGAGTGAGGAAGGAGGA
VEGFR2 R	GGATGATGACAAGAAGTAGCCAGAA
NRP1 F	GGAAACACCAACCCCACAGA
NRP1 R	CATACCCAACATTCCAGAGCAAG
TGFB1 F	CCTGGCGATACCTCAGCAAC
TGFB1 R	GCTAAGGCGAAAGCCCTCAA
PECAM1 F	CAGGACCGCGTTTTATCCTTC
PECAM1 R	TGATGTGGAACTTGGGTGTAGAGA
DLL1 F	GAGCGTGGGGAGAAAGTGTG
DLL1 R	ACTTGCATTCCCCTGGTTTGT
DLL4 F	ACCCTCTCCAACTGCCCTTC
DLL4 R	TGCTGGTTTGCTCATCCAATAAC
JAG1 F	CAGATTTCCTTGTTCCCTTGCT
JAG1 R	CGTTGTTGGTGGTGTTGTCCT
NOTCH1 F	CGACAACGCCTACCTCTGCT
NOTCH1 R	ACAGGCACACTCGTAGCCATC
RBPJK F	TACGAGTGTGGTTTGGGGATG
RBPJK R	GTAGGTAAAGGTAAGGCTGGTGGAA
PROX1 F	GGCTCTCCTTGTCGCTCATAAA

PROX1 R	GGAGCTGGGATAACGGGTATAAAAA
NR2F2 F	AGTGGGCATGAGACGGGAAG
NR2F2 R	GACAGGTACGAGTGGCAGTTGAG
SOX18 F	TCATGGTGTGGGCAAAGGAC
SOX18 R	GTTCAGCTCCTTCCACGCTTT
FOXC1 F	GGAGATGTTCGAGTCACAGAGGA
FOXC1 R	GACGTGCGGTACAGAGACTGG
FOXC2 F	GAGTCCCAGGTGAGTGGCAAT
FOXC2 R	ATTTCGTGCAGTCGTAGGAGTAGG
ERG F	CCAGCGTCCTCAGTTAGATCCTT
ERG R	CATCTTGAACTCCCCGTTGGT
ETV2 F	CGATGCCCCAAAACTAACCA
ETV2 R	TAATTCATGCCCGGCTTTCTC
MEF2C F	CAGGACAAGGAATGGGAGGA
MEF2C R	ACTGACTGAGGGCAGATGGTG
ATX F	AGAGCAGAAGGATGGGAGGAAG
ATX R	TCACAGCGACAATCAGGAGGT
HEY1 F	AGGTTCCATGTCCCCAACTACA
HEY1 R	TGCAGGATCTCGGCTTTTTC
HEY2 F	CGTCGGGATCGGATAAATAACA
HEY2 R	CAAGAGCGTGTGCGTCAAAG

Supplementary Table3 qPCR primers for mouse

**Primer Name**	**Primer sequence 5'-3'**
Shp2 F	CAACGACGGCAAGTCCAAAG
Shp2 R	AGAGAGTCAAAGCGCTCTCC
Sox7 F	ATGCTGGGAAAGTCATGGAAG
Sox7 R	CGTGTTCTGGTCACGAGAGA
Vegfa F	GGAGATCCTTCGAGGAGCACTT
Vegfa R	GGCGATTTAGCAGCAGATATAAGAA
Fgf-1 F	GACTTCATTCCCGTCTTGTG
Fgf-1 R	TAGTTTCCTAGAGGCAGGTT
Fgf2 F	CACGTCAAACTACAACTCCA
Fgf2 R	CGTCCATCTTCCTTCATAGC
Gapdh F	CTTCACCACCATGGAGGAGGC
Gapdh R	GGCATGGACTGTGGTCATGAG
