# Supplementary material for: Endothelial deletion of SHP2 suppresses tumor angiogenesis and promotes vascular normalization
Source: Nat Commun. 2021 Nov 2;12:6310. doi: 10.1038/s41467-021-26697-8 (PMC8564544; doi:10.1038/s41467-021-26697-8)
Supplement: Supplementary file 3 — Reporting Summary [file 41467_2021_26697_MOESM3_ESM.pdf]

nature research

Corresponding author(s): Hongqiang Cheng

Last updated by author(s): Sep 24, 2021

Reporting Summary

Nature Research wishes to improve the reproducibility of the work that we publish. This form provides structure for consistency and transparency in reporting. For further information on Nature Research policies, see our Editorial Policies and the Editorial Policy Checklist.

Statistics

For all statistical analyses, confirm that the following items are present in the figure legend, table legend, main text, or Methods section.

n/a	Confirmed	
☐	☒	The exact sample size (*n*) for each experimental group/condition, given as a discrete number and unit of measurement
☐	☒	A statement on whether measurements were taken from distinct samples or whether the same sample was measured repeatedly
☐	☒	The statistical test(s) used AND whether they are one- or two-sided<br>*Only common tests should be described solely by name; describe more complex techniques in the Methods section.*
☐	☒	A description of all covariates tested
☐	☒	A description of any assumptions or corrections, such as tests of normality and adjustment for multiple comparisons
☐	☒	A full description of the statistical parameters including central tendency (e.g. means) or other basic estimates (e.g. regression coefficient) AND variation (e.g. standard deviation) or associated estimates of uncertainty (e.g. confidence intervals)
☐	☒	For null hypothesis testing, the test statistic (e.g. *F*, *t*, *r*) with confidence intervals, effect sizes, degrees of freedom and *P* value noted<br>*Give P values as exact values whenever suitable.*
☒	☐	For Bayesian analysis, information on the choice of priors and Markov chain Monte Carlo settings
☒	☐	For hierarchical and complex designs, identification of the appropriate level for tests and full reporting of outcomes
☒	☐	Estimates of effect sizes (e.g. Cohen's *d*, Pearson's *r*), indicating how they were calculated

Our web collection on statistics for biologists contains articles on many of the points above.

Software and code

Policy information about availability of computer code

Data collection	CFX96 Touch Real-Time PCR Detection System (Bio-Rad); odyssey (version 3.0.29) and LI-COR image studio(version 5.2); IX70 inverted microscope(Olympus) ; Olympus VS200 (Olympus); Amaxa Nucleofector II (Amaxa);mPromega GloMax 20/20 (Promega), SynergyMx M5 (Molecular Devices).
Data analysis	GraphPad Prism v6.0 and v8.0; ImageJ 1.49v; OlyVIA VS200(Olympus)

For manuscripts utilizing custom algorithms or software that are central to the research but not yet described in published literature, software must be made available to editors and reviewers. We strongly encourage code deposition in a community repository (e.g. GitHub). See the Nature Research guidelines for submitting code & software for further information.

Data

Policy information about availability of data

All manuscripts must include a data availability statement. This statement should provide the following information, where applicable:

- Accession codes, unique identifiers, or web links for publicly available datasets
- A list of figures that have associated raw data
- A description of any restrictions on data availability

Single cell RNA sequencing dataset (GSE118904) used in Figure 1d, supplemental Figure 7f-g were freely available from reference 38. Source data for all figures will be included in supplemental materials after the manuscript accepted.

Field-specific reporting

Please select the one below that is the best fit for your research. If you are not sure, read the appropriate sections before making your selection.

☒ Life sciences ☐ Behavioural & social sciences ☐ Ecological, evolutionary & environmental sciences

For a reference copy of the document with all sections, see nature.com/documents/nr-reporting-summary-flat.pdf

Life sciences study design

All studies must disclose on these points even when the disclosure is negative.

Sample size	Sample size estimates has been performed on previous experience to obtain statistical significance and reproducibility. For in vitro experiments such as Western blot, qPCR, IHC, CO-IP and ubiquitination assay, at least three samples were used per group for minimal statistics requirements. For in vivo studies, the sample size was determined to be sufficient to obtain the statistical difference between groups.
Data exclusions	No data were excluded.
Replication	Every experiment was replicated at least two to three independent experiments with similar results and the number of repeats is stated in individual figure legends.
Randomization	Animals were randomized into the experimental groups with equivalent body weight. For cell experiments, randomization was not relevant, because cells were prepared and treated in the same way by the same researchers regardless of the treatments. For NSCLC tumor tissues and paired adjacent normal tissues, sample were randomized. For NSCLC tumor tissues and paired adjacent normal tissues, the researcher groups the samples based on sample information without bias.
Blinding	The investigators were not blinded to group allocation during data collection and/or analysis, because mice needed to be genotyped by PCR and cells were treated by the researchers who collected the data. No specific blinding was applied since all experiments were assigned into groups including relevalant controls and analysis was done objectively and without bias.

Reporting for specific materials, systems and methods

We require information from authors about some types of materials, experimental systems and methods used in many studies. Here, indicate whether each material, system or method listed is relevant to your study. If you are not sure if a list item applies to your research, read the appropriate section before selecting a response.

Materials & experimental systems

n/a	Involved in the study	
☐	☒	Antibodies
☐	☒	Eukaryotic cell lines
☒	☐	Palaeontology and archaeology
☐	☒	Animals and other organisms
☐	☒	Human research participants
☒	☐	Clinical data
☒	☐	Dual use research of concern

Methods

n/a	Involved in the study	
☒	☐	ChIP-seq
☒	☐	Flow cytometry
☒	☐	MRI-based neuroimaging

Antibodies

Antibodies used	Western blotting:<br>Rabbit anti-ASK1(1:1000; Novus Biologicals NB100-81788)<br>Rabbit anti-SHP2(1:1000; Cell Signaling Technology #3397)<br>Rabbit anti-p-SHP2(1:1000; Abcam ab62322)<br>Rabbit anti-SOX7(1:1000; Proteintech 23925-1-AP)<br>Rabbit anti-SOX17(1:1000; Proteintech 24903-1-AP)<br>Rabbit anti-p-c-Jun S63(1:1000; Cell Signaling Technology #91952)<br>Rabbit anti-p-c-Jun S73(1:1000; Cell Signaling Technology #3270)<br>Rabbit anti-c-Jun(1:1000; Diagbio db2622)<br>Rabbit anti-p-ERK(1:1000; Cell Signaling Technology #9101)<br>Rabbit anti-ERK(1:1000; Cell Signaling Technology #4695)<br>Rabbit anti-p-p38(1:1000; Cell Signaling Technology #4511)<br>Rabbit anti-p38(1:1000; Cell Signaling Technology #8690)<br>Mouse anti-β-Actin(1:1000; Huabio M1210-2)<br>Rabbit anti-Vegfa(1:1000; Proteintech 19003-1-AP)<br>Mouse anti-HA(1:1000; Beyotime AF5057)

Mouse anti-MYC(1:1000; Beyotime AF5054)
Goat anti-Rabbit(1:5000; LI-COR 925-32211)
Goat anti-Mouse(1:5000; LI-COR 925-68070)
Immunofluorescence :
Rabbit anti-ASK1(1:100 ; Novus Biologicals NB100-81788)
Mouse anti-SHP2(1:50; Santa Cruz sc-7384)
Rabbit anti-p-SHP2(1:100; Abcam ab62322)
Rabbit anti-SOX7(1:100 ; Abcam ab220293)
Rabbit anti-p-c-Jun S63(1:100 ; Cell Signaling Technology #91952)
Rabbit anti-p-c-Jun S73(1:100 ; Cell Signaling Technology #3270)
Goat anti-CD31(1:100 ; R&D system AF3628)
Rabbit anti-CD31(1:100 ; Abcam ab28364)
Rabbit anti-collagen IV(1:100 ; Abcam ab236640)
Rabbit anti-α-SMA(1:100 ; Cell Signaling Technology #19245)
Rabbit anti-CD11b(1:100 ; Biolegend 101213)
FITC conjugated mouse anti-pimonidazole monoclonal antibody(1:100;Hypoxyprobe;HP1-100Kit)
Donkey anti-Mouse, Alexa Fluor 488(1:200 ; ThermoFisher A-21202)
Donkey anti-Rabbit, Alexa Fluor 488(1:200 ; ThermoFisher A-21206)
Donkey anti-Rabbit, Alexa Fluor 555(1:200 ; ThermoFisher A-31572)
Donkey anti-Goat, Alexa Fluor 647(1:200 ; ThermoFisher A-21447)
AffiniPure Donkey Anti-Goat, Alexa Fluor 488(1:200 ; Jackson Immuno Research 705-545-103)
AffiniPure Donkey Anti-Goat, Cyanine Cy3(1:200 ; Jackson Immuno Research 705-165-103)

Validation

All of the antibodies used in this study are commercially available and validated for the application and species by the manufacturers, whose data are described at the manufacturer's website.

Rabbit anti-ASK1(NB100-81788):
Reactivity: human, mouse, rat
Application: WB, IHC,IF

Rabbit anti-SHP2(#3397)
Reactivity: human, mouse, rat
Application: WB, IHC,IP

Rabbit anti-p-SHP2(ab62322)
Reactivity: human, mouse
Application: WB, ICC,IP,IF

Rabbit anti-SOX7(23925-1-AP)
Reactivity: human, mouse
Application: WB, IHC, IF, ELISA

Rabbit anti-SOX7(ab220293)
Reacts with: Human,Mouse, Rat
Application: WB, IHC-P

Rabbit anti-SOX17(24903-1-AP)
Reactivity: human, mouse, rat
Application: WB, ELISA

Rabbit anti-p-c-Jun S63(#91952)
Reactivity: human, mouse, rat
Application: WB, IF, ChIP, FC, IP

Rabbit anti-p-c-Jun S73(#3270)
Reactivity: human, mouse, rat
Application: WB, IF, ChIP, FC, IP, IHC

Rabbit anti-c-Jun(db2622)
Reactivity: human, mouse, rat
Application: WB, IF, IP, IHC,ICC

Rabbit anti-p-ERK(#9101)
Reactivity: human, mouse, rat
Application: WB, IF, IP, FC

Rabbit anti-ERK(#4695)
Reactivity: human, mouse, rat
Application: WB, IF, IP, FC, IHC

Rabbit anti-p-p38(#4511)
Reactivity: human, mouse, rat
Application: WB, IF, IP, FC, IHC

Rabbit anti-p38(#8690)
Reactivity: human, mouse, rat
Application: WB, IF, FC, IHC

Mouse anti-β-Actin(M1210-2)
Reactivity: human, mouse, rat
Application: WB, ICC, FC, IHC

Rabbit anti-Vegfa(19003-1-AP)
Reactivity: human, mouse, rat
Application: RIP, IHC, IF, CoIP,ELISA

Mouse anti-HA(AF5057)
Reactivity: human, mouse, rat
Application: WB, IP

Mouse anti-MYC(AF5054)
Reactivity: human, mouse, rat
Application: WB, IP, IF

Mouse anti-SHP2(sc-7384)
Reactivity: human, mouse, rat
Application: WB, IP, IF, ELISA, IHC, ICC

Goat anti-CD31(AF3628)
Reactivity: mouse, rat
Application: WB, IP, IF, IHC, ICC

Rabbit anti-CD31(ab28364)
Reactivity: human, mouse
Application: IHC

Rabbit anti-collagen IV(ab236640)
Reactivity: human, mouse, rat
Application: IHC

Rabbit anti-α-SMA(#19245)
Reactivity: human, mouse, rat
Application: WB, IP, IF, IHC

Rabbit anti-CD11b(101213)
Reactivity: human, mouse, rat
Application: WB, IP, IF, IHC,FC
Immunofluorescent staining and western blots showed relatively little background noise and proteins were detected at expected kDa sizes on western blots.

Eukaryotic cell lines

Policy information about cell lines

Cell line source(s)	293T, H460, A549, H1299, LM3, LLC, E0771, B16 cells were obtained from ATCC. Human cerebral microvessel endothelial cells (hCMECs) were purchased from Zhejiang Meisen Cell Technology Co., Ltd. HUVEC cells were isolated from normal human umbilical veins, which were collected from Women's Hospital, Zhejiang University School of Medicine. MLEC cells were isolated from Shp2f/f and Shp2f/f:CDH5-CreERT2 (SHP2iECKO) mouse lung tissues.All mice were cultured in suitable temperature and humidity environment and fed with sufficient water and food. (25 ℃, suitable humidity (typically 50%), 12 hour dark/light cycle)
Authentication	The cell lines were not authenticated.
Mycoplasma contamination	All cell lines were validated to be free of mycoplasma contamination.
Commonly misidentified lines (See ICLAC register)	No commonly misidentified cell lines were used.

Animals and other organisms

Policy information about studies involving animals; ARRIVE guidelines recommended for reporting animal research

Laboratory animals	Shp2f/f and Shp2f/f:CDH5-CreERT2 (SHP2iECKO) were originally generated in our laboratory as reported previously(FASEB J 2019

Laboratory animals	33(1):1124-37).All of the in vivo experiments were performed in C57BL/6J background mice, and Shp2f/f were used as controls. 8 week-old male mice were used in most experiments. All mice were maintained with free access to food and water in SPF condition.
Wild animals	This study did not involve wild animals.
Field-collected samples	This study did not involve field-collected samples.
Ethics oversight	All animal protocols were approved by the Animal Care and Use Committee of the Zhejiang University School of Medicine

Note that full information on the approval of the study protocol must also be provided in the manuscript.

Human research participants

Policy information about studies involving human research participants

Population characteristics	NSCLC tissues and matched tumor-adjacent normal lung tissues were obtained from 9 patients (median age: 64 years old, range from 52 to 78; male: 6, female: 4).
Recruitment	All cancer patients volunteers were recruited without bias from the First Affiliated Hospital of Zhejiang University.Written informed consent was obtained from all patients prior to the study.
Ethics oversight	This study was approved by the First Affiliated Hospital of Zhejiang University School of Medicine Ethics Committee

Note that full information on the approval of the study protocol must also be provided in the manuscript.
